# Crucial Convolution: Genetic and Molecular Mechanisms of Coiling during Epididymis Formation and Development in Embryogenesis

**DOI:** 10.3390/jdb10020025

**Published:** 2022-06-14

**Authors:** Joanne Wong, Jemma Gasperoni, Jarrad Fuller, Sylvia V. H. Grommen, Bert De Groef, Cathryn Hogarth, Sebastian Dworkin

**Affiliations:** 1 Department of Microbiology, Anatomy, Physiology and Pharmacology, La Trobe University, Melbourne, VIC 3086, Australia; joannewong001@gmail.com (J.W.); 17169275@students.latrobe.edu.au (J.G.); 19327947@students.latrobe.edu.au (J.F.); sylvia.grommen@kuleuven.be (S.V.H.G.); bert.degroef@kuleuven.be (B.D.G.); 2 Department of Rural Clinical Science, La Trobe Rural Health School, La Trobe University, Wodonga, VIC 3690, Australia; c.hogarth@latrobe.edu.au; 3 School of Agriculture, Biomedicine and Environment, La Trobe University, Melbourne, VIC 3086, Australia

**Keywords:** coiling, development, epididymis, male infertility, male reproductive tract, tubulogenesis, Wolffian duct

## Abstract

As embryonic development proceeds, numerous organs need to coil, bend or fold in order to establish their final shape. Generally, this occurs so as to maximise the surface area for absorption or secretory functions (e.g., in the small and large intestines, kidney or epididymis); however, mechanisms of bending and shaping also occur in other structures, notably the midbrain–hindbrain boundary in some teleost fish models such as zebrafish. In this review, we will examine known genetic and molecular factors that operate to pattern complex, coiled structures, with a primary focus on the epididymis as an excellent model organ to examine coiling. We will also discuss genetic mechanisms involving coiling in the seminiferous tubules and intestine to establish the final form and function of these coiled structures in the mature organism.

## 1. Introduction

### 1.1. The Need for and the Mechanisms of Coiling and Folding across Development

During embryonic development, numerous organs and tissues undergo substantial coiling, folding or looping in order to fit more surface area of tissue into a defined volume of available cavity. Notable examples include the small intestine, the proximal and distal tubules of the nephron within the kidney [1], the epididymis [2] and seminiferous tubules [3], the cochlea [4] and the midbrain–hindbrain boundary (MHB) in teleost fishes [5]. Coiling allows these organs a greater capacity for function, for example a longer duration during which essential nutrients and other compounds can be resorbed from filtered blood (kidney) or digested food (intestine), the production and “assembly-like” maturation of sperm in the seminiferous tubules and epididymis, and the capacity for greater direct neuron–neuron contact in the MHB. Defects in coiling can lead to serious developmental abnormalities and defects in adult life. Improper cardiac looping is a prominent cause of congenital cardiac malformations [6], and the misfolding of the epididymis underpins many cases of male infertility [7] and aberrant gut looping can result in obstruction and intestinal malrotation [8]. Therefore, the establishment of properly folded organs during embryogenesis is an important hallmark of normal development.

The development of these coiling/folding systems has allowed for greater complexity in evolution, with the development of the most highly coiled organs and tissues typically seen in higher mammals such as primates. Somewhat unsurprisingly, many molecular pathways that govern tissue folding and coiling are well conserved from flies to humans, and we will review some of these below.

In this review, we analyse the current state of knowledge governing genetic and developmental mechanisms that underpin correct coiling/folding, with a particular focus on epididymal establishment and function and a brief overview of coiling in other tissues. Many of the mechanistic regulators of epididymal coiling have been uncovered using transgenic rodent models. In this respect, the epididymis is not alone—numerous other tubular structures are similarly present with boundaries defined by the tight expression of transcription factors such as rhombomeres in the hindbrain [9], seminiferous tubules of the testes [10] and regions within the tubules of the kidney [11]. In fact, it appears clear that transcriptional demarcation, at least in mammals, may be a conserved hallmark in all tubular structures, governing embryonic development, coiling during maturation and/or subsequent function in the adult.

#### 1.1.1. The Epididymis as a Model for Understanding Tissue Coiling

A key developmental event is the convolution of coiled tubules, which is required for achieving the correct duct length and diameter, and ultimately full functionality. The importance of ductal coiling in the developing epididymis is becoming more apparent, as numerous knockout (KO) rodent models that exhibit abnormal coiling also present with impaired fertility. A number of reviews have summarised the current knowledge and proposed mechanisms of tubulogenesis but do not discuss the mechanisms of tubule coiling [12,13,14]. A number of studies have begun to uncover the mechanisms, genes and pathways involved in gut [8,15,16,17,18] and heart looping [19,20,21,22,23,24,25]; however, those behind the intense coiling of the epididymal tube remain elusive.

The epididymis is a long, segmented and highly convoluted tube that connects the testis (the site of sperm production) to the vas deferens (the passage by which mature, functional sperm capable of fertilisation are released into the ejaculatory duct). The epididymal maturation of spermatozoa is critical for male fertility, yet the mechanisms that govern the development and function of this essential male reproductive organ remain understudied. Although traditional anatomical segmentation of this structure has broadly divided it into three distinct regions (the caput (also comprising the initial segment), corpus and cauda), recent work has elegantly demonstrated that the mammalian epididymis is instead neatly subdivided into 10–19 disparate segments (species-dependent), with tightly defined, transcriptionally demarcated boundaries. Together with the vas deferens and seminal vesicles, the epididymis originates from the Wolffian (mesonephric) duct, a tubule embryologically derived from the intermediate mesoderm [26].

The adult epididymal tubule is just over 1 m long in mice and 6 m long in humans [2], and sperm transit through the duct takes between 5.5 and 10 days depending on the species [27]. During this time, sperm are biochemically and structurally modified to produce functionally mature sperm capable of motility and fertilisation of the oocyte. Proteins secreted by the epididymal epithelium (many of which have been determined by, e.g., mass spectrometry [28]) are largely responsible for these modifications, as spermatozoa themselves are transcriptionally inactive [7,29].

**Figure 1:**
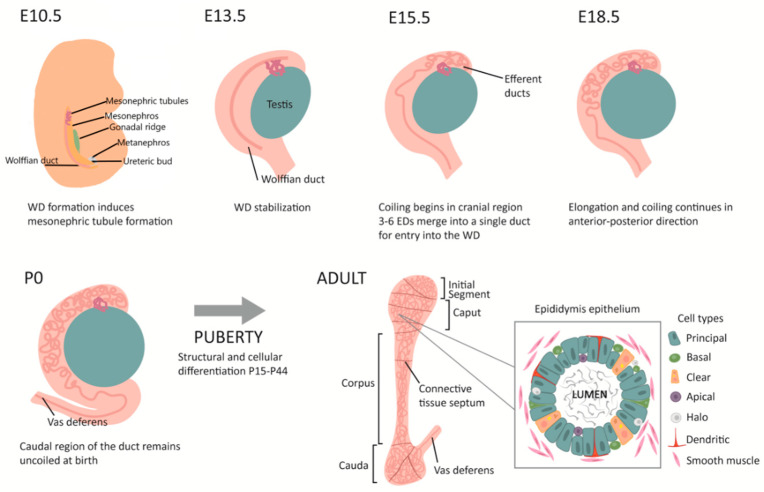
Schematic diagram of Wolffian and epididymal duct development in mouse. ED: efferent ducts; WD: Wolffian duct.

The Wolffian duct has its origins as a straight tubule, only millimetres in length, that must undergo an incredible transformation during maturation to establish a fully functional organ. Coiling is essential in achieving the appropriate tubule length and segmentation required for sperm maturation within the constraints of the diminutive space of the epididymis. The proximal mesenchymal tissue of the duct undergoes extensive remodelling and coiling during embryonic and postnatal growth to become a fully differentiated epididymis by puberty (Figure 1) [30,31].

**Figure 2:**
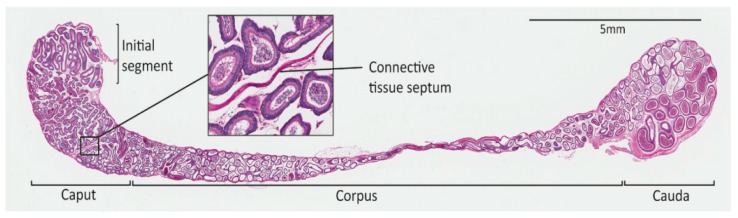
Section of an adult mouse epididymis. Gross segments are indicated and a close-up of a connective-tissue septum is shown.

In the mouse, Wolffian duct coiling begins at E14 in one plane in the proximal region, and caudally progresses as development continues. Three-dimensional coiling and folding begins to occur from E16 in the initial segment and caput [30]. After birth, the epididymal duct goes through a significant transformation involving duct elongation and convolution in the cauda region [32]. Finally, at the onset of puberty, the epithelium undergoes significant structural changes and pseudostratification to form the fully developed adult epididymis [32,33,34] (Figure 2). This section will now focus on our current understanding of the factors involved in epididymal coiling process.

It is notable that in studies of aberrant Wolffian duct and epididymis coiling, cell proliferation is usually investigated as the underlying cause. However, cell proliferation is only one of many aspects of tubulogenesis and tissue remodelling during development. Such studies are likely to benefit from a broader investigation of cellular developmental processes, including an analysis of other mechanisms involved in tubular morphogenesis such as cell migration, cell rearrangements, cell adhesion and the remodelling of the extracellular matrix (ECM) [5,6,7].

Paracrine signalling is likely to be a significant part of what is sure to be a complex signalling system, facilitating some areas of the duct to receive “coil” signals and others to receive “do-not-coil” signals [30]. The vas deferens, connecting the epididymis to the ejaculatory duct, is a continuation of the epididymal duct, yet strict regulation of signalling ensures that the vas deferens does not coil [30]. It has also been suggested that in species with septa dividing segments of the epididymis, the septa contribute to isolating paracrine signalling to a specific segment [33,35], and that these paracrine signalling changes will likely correlate with the altered expression and activity of upstream transcription factors [34]. However, the human epididymis lacks well-formed connective tissue septa, yet the epididymis is still able to convolute in this species, alluding to an alternative or additional mechanism.

#### 1.1.2. Androgen Signalling

In addition to their role in sexual differentiation, masculinisation and male reproductive tract development, androgens are crucial for the transformation of the short, straight Wolffian duct into a long, coiled epididymis. The importance of androgen-controlled mesenchymal–epithelial interactions and of mesenchymal androgen receptors (ARs) in epithelial development and function in the epididymis has been well documented (for reviews, see [36,37,38]). However, the role of ARs in the epithelium and in some aspects of epididymis development is less clear, particularly as KO models indicate that androgens are only required at some stages of certain developmental events [39].

In utero treatment of pregnant rats with a combination of flutamide (an AR antagonist) and di(*n*-butyl) phthalate (DBP; an inhibitor of foetal testosterone synthesis) showed that Wolffian duct stabilisation is not affected by the inhibition of androgen signalling; normal Wolffian ducts were observed at E18.5 and E19.5 in treated animals [40]. However, later in embryonic development, Wolffian duct differentiation was impaired, as evidenced by abnormal morphology, the absence of corpus segments, reduced coiling and elongation of the duct at E21.5, and underdeveloped Wolffian duct-derived structures in adult males. Flutamide/DBP treatment also resulted in decreased numbers of proliferating epithelial and stromal cells in the Wolffian duct at E19.5–E21.5 [40]. While it is recognised that the flutamide/DBP treatment of pregnant animals is unlikely to abolish all androgen signalling in the embryos, these data indicate the possibility of a non-androgenic mechanism that controls or contributes to Wolffian duct stabilisation. They also show that later in epididymis development, androgens are required for normal coiling. However, even in coiling, a non-androgenic control mechanism may contribute: in the same study, female rats exposed to testosterone in utero showed a stabilised Wolffian duct at E18.5, and although this did not develop into an epididymis, some duct coiling was observed at E21.5, but not to the same extent as in control males [41]. The fact that testosterone treatment in females could not completely mimic the coiling occurring in males likely indicates that there are factors other than testosterone that contribute to duct coiling.

**Figure 3:**
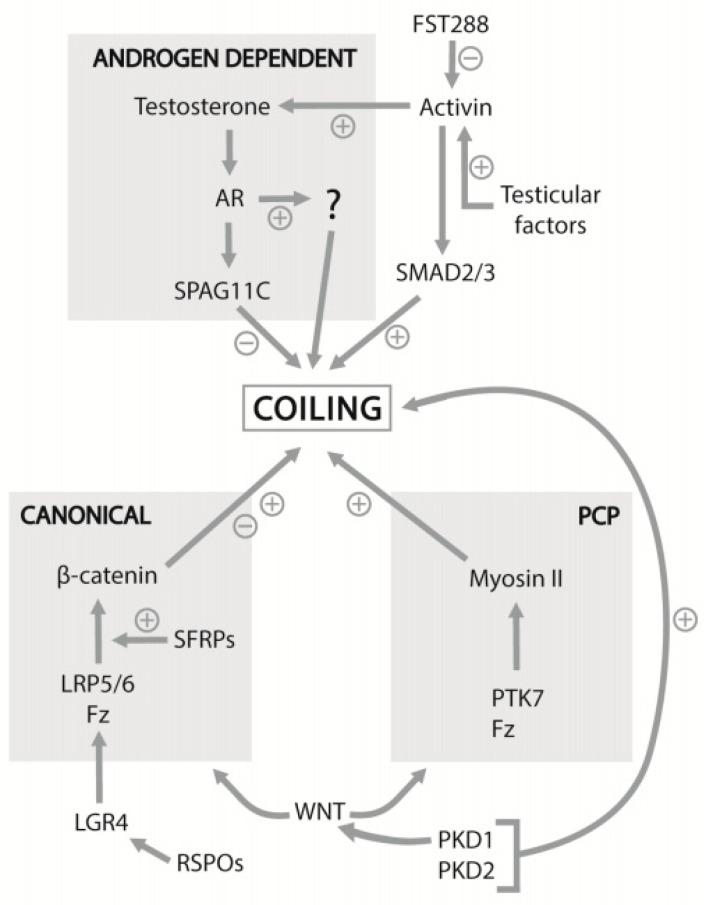
Overview of interactions between the genes, proteins and pathways implicated in epididymis coiling discussed in this review. Additional interactions may exist between these factors. RSPOs: R-spondins; Fz: frizzled receptor; LRP: lipoprotein receptor-related protein. For other abbreviations, see main text.

Androgens indeed play an important role as regulators of other factors involved in epididymal coiling. Sperm-associated antigen 11c (SPAG11C) is an androgen-regulated β-defensin whose expression has been inversely correlated with coiling [42]. SPAG11C belongs to a group of antimicrobial proteins secreted from the epididymal epithelium that are suspected to play a role in male fertility in rodents and humans via their involvement in sperm maturation and function [43,44,45,46], potentially via interaction with the sperm head [47]. In the embryonic rat Wolffian duct, a drop in *Spag11c* mRNA expression levels coincided with an increase in circulating testosterone levels and the onset of Wolffian duct coiling [42,48]. Furthermore, rat Wolffian ducts cultured in medium containing purified human recombinant SPAG11C failed to elongate, duct coiling was decreased, and the proliferation of epithelial cells was dramatically lowered compared to wild types (WTs) [42]. Coiling was partially restored when SPAG11C was removed from the medium. Evidently, SPAG11C inhibits, while testosterone stimulates, epithelial cell proliferation, the latter at least in part through the suppression of SPAG11C expression (Figure 3). As testosterone regulates SPAG11C expression, it likely contributes to the control of the “coil” and “do-not-coil” signals that coordinate the coiling of the duct. The generation of a *Spag11c* KO mouse line would be useful to further elucidate the role of SPAG11C in Wolffian duct coiling, and the investigation of the contribution of other SPAG11 isoforms to epididymis development may be worthwhile.

In contrast to the global KO of ARs, studies showed that the epithelium-specific ablation of ARs did not affect Wolffian duct epithelium cell proliferation, survival nor duct elongation and coiling [26]. Compared to controls, increased apoptosis was observed in the Wolffian duct epithelia of global ARKOs but not in epithelium-specific ARKOs [49]. Stabilised and coiled Wolffian ducts were observed at E18.5 in epithelium-specific ARKOs, whereas in global ARKO males, at least in this study, the Wolffian duct started to regress four days earlier at E14.5. These data indicate that while epithelial ARs are dispensable for inhibiting cell death in males, mesenchymal ARs are not, and that Wolffian duct coiling can persist in the absence of epithelial ARs. However, the differentiation of basal cells in the caput and of principal cells in the caput and cauda was diminished in epithelium-specific ARKOs, showing a requirement for epithelial androgen signalling in the differentiation of some cell types [49].

#### 1.1.3. WNT Signalling

In addition to androgen, various non-steroidal regulators of duct coiling have recently been revealed (Figure 3). One of those is WNT signalling, known to be involved in reproductive tract and kidney development [50]. *Wnt9b* KO mice exhibited a complete lack of epididymides at birth, even though developing Wolffian ducts were observable at E10.5–E11.5 [51]. Spermatozoa are WNT-signalling responsive; WNT signals released from the epididymal epithelium are required for normal sperm tail structure and motility, and are therefore crucial for sperm maturation [52]. In the Wolffian duct, WNT signalling only occurs in the epithelium, and in the adult epididymis, it is largely restricted to the epithelium of the second to fourth segments of the caput [53]. It has recently been demonstrated that canonical WNT signalling is required for normal Wolffian duct morphogenesis, as the pharmacological inhibition of the WNT signalling pathway resulted in decreased duct coiling, decreased cell proliferation and increased apoptosis in cultured Wolffian ducts [53]. In the same study, high β-catenin expression was shown in the Wolffian duct epithelium, and the epithelium-specific deletion of the β-catenin gene resulted in the absence of coiling in the epididymides from 1-day-old pups [53]. The overexpression of β-catenin, causing the constitutive activation of WNT signalling, also resulted in straight epididymal ducts at birth, in combination with increased cell proliferation and apoptosis, indicating that the correct expression level of β-catenin is required for proper duct coiling to occur [53]. WNT target gene analysis has shown that the fibroblast growth factor 7 gene (*Fgf7*), whose gene product promotes WNT signalling by blocking the WNT inhibitor Dickkopf-related protein 1, is a target gene of WNT [53]. Moreover, FGF7 is expressed in the epithelium and mesenchyme of the Wolffian duct, and cultured ducts treated in vivo with FGF7 showed increased coiling [53]. A study on epithelium-specific fibroblast growth factor receptor 2 KO mice demonstrated that epithelial FGF signalling is required in the Wolffian duct to regulate cell proliferation and prevent regression specifically in the caudal region of the duct [54]. Notably, WNT signalling was not affected when androgen signalling was inhibited in cultured Wolffian ducts, indicating that this pathway in the Wolffian duct is not dependent on androgen signalling [53].

Furthermore, secreted Frizzled-related proteins, encoded by *Sfrp* genes, inhibit WNT signalling via their ability to bind to the WNT receptor Frizzled. Male mice lacking both *Sfrp1* and *Sfrp2* genes exhibited decreased duct coiling at E17.5 compared to WT controls, and testosterone levels were not affected in E14.5 mutants [55]. These double mutants also exhibited a range of gonadal abnormalities, with a similar phenotype to *Wnt5a* KOs, leading the authors to suggest that *Sfrp* genes may positively regulate WNT signalling in the epididymis rather than inhibit it.

Finally, conditional KOs specifically lacking *Wnt5a* expression in the primary mesoderm exhibited thickened and duplicated Wolffian ducts [56]. This hypercellularity is potentially due to defective WNT signalling in the non-canonical planar cell polarity (PCP) pathway, irregularities in which are known to result in improper cell orientation during division and migration, and anterior–posterior body axis extension [56]. Other genes involved in the PCP pathway have also been implicated in decreased Wolffian duct coiling. For example, in protein tyrosine kinase 7 (*Ptk7*) conditional KO mice, in which *Ptk7* was knocked out in the mesoderm only, the Wolffian duct of E18.5 mice was shorter and wider compared to controls, coiling was significantly reduced, and sperm motility was severely decreased [57]. The mitotic spindle was not primarily orientated along the elongation axis, indicating that cell division is not the major mechanism for tube elongation (as it is in the kidneys). The authors analysed the duct lumen diameter, orientation of cell division, epithelial cell shape, epithelium PCP and orientated cell rearrangements, leading to the findings that there was a loss of PCP characteristics and impaired convergent extension in the developing Wolffian duct epithelium, resulting in decreased duct elongation and coiling. 

Testosterone supplementation in Wolffian duct culture did not restore duct elongation and coiling. However, the cell proliferation and orientation of cell division was not affected in mutants, leading to the hypothesis that PTK7 is involved in duct elongation via its regulation of convergent extension-like cell rearrangement rather than cell proliferation, allowing the duct to elongate and coil without changing the luminal diameter. Convergent extension is the restructuring of an embryonic tissue based on cellular movement, in which the cells converge along one axis, thereby extending (elongating) the tissue along a perpendicular axis. This process is dependent on PCP mechanisms, which are also required for the development of other organs such as the kidney, heart and pancreas [58] and is known to be controlled by PTK7 via its regulation of myosin II [59,60]. Indeed, myosin II inhibition by blebbistatin in Wolffian duct culture resulted in the same phenotype as conditional *Ptk7* KOs [57].

#### 1.1.4. Activin A

Activin A and inhibins are paracrine factors from the TGF-β superfamily with an established role in male reproductive tract development and function [61,62,63,64], hormone signalling [65], immunity and inflammation [66,67], and spermatogenesis [68,69]. Inhibin β_A_ (INHBA) is an activin A subunit, known to be a negative regulator of branching tubulogenesis in the kidneys [70,71]. In addition, the *Inhba* gene was shown to be required for the coiling of the anterior Wolffian duct (Figure 3), and is strongly expressed in this region compared to the posterior Wolffian duct, which gives rise to the vas deferens [72]. Initial INHBA action begins before testosterone production, as *Inhba* expression was observed at E12.5 [72]. At E12.5, E17.5 and E19.5, *Inhba* expression was restricted to the mesenchyme, while phospho-SMAD2/3 (an activin effector) was exclusively expressed in epithelial cells, indicating that activins are mesenchyme-derived paracrine factors [72]. Activins (but not inhibins) act via the SMAD2/3 pathway, so the expression of phospho-SMAD2/3 in the epithelial nuclei indicates that the activin—rather than the inhibin—pathway is active in the Wolffian duct. *Inhba* expression at E12.5 showed the same expression pattern in mesonephroi from both male and female embryos, providing further evidence that early *Inhba* expression is not testosterone-dependent [72]. Furthermore, at E13.5, mesonephroi cultured in the absence of testicular tissue exhibited reduced *Inhba* expression, which testosterone supplementation could not fully restore. Mesonephroi cultured with the testes attached showed normal *Inhba* expression, indicating that other testicular factors, in addition to testosterone, play a role in regulating *Inhba* expression from E13.5 onwards. In E17.5–E19.5 *Inhba*-deficient embryos, the Wolffian duct failed to elongate and coil [72]. The Wolffian duct in these embryos appeared developmentally stalled, remaining morphologically similar to E15.5 control mice. Testosterone production was shown to be normal in *Inhba*-deficient embryos, further indicating that the lack of coiling is unlikely to be the result of decreased testosterone levels. Epithelial cell proliferation failed to increase from E17.5 to E19.5 in KOs as it did in WT mice, which likely contributes to the lack of duct elongation and coiling [72].

In 8-week-old *Inhba^+/−^* mice, which showed a 70% decrease in serum activin A (dimers of INHBA) levels and a 50% decrease in epididymal *Inhba* mRNA and protein levels compared to WTs, the morphology and the transcriptome of the epididymis was normal [73,74]}. These observations suggest that low levels of activin A (or INHBA) are sufficient for normal epididymis development. This is possibly because the role of activin A can be to some extent overtaken by activin B and dimers of inhibin β_B_ (INHBB) [73,74]. Like *Inhba*, *Inhbb* is mainly expressed in the caput epididymis. In mice in which both *Inhba* alleles were replaced with *Inhbb* sequences, epididymis development was delayed at 6 weeks of age, but by 8 weeks, epididymis morphology was indistinguishable from WTs [73,74].

**Figure 4:**
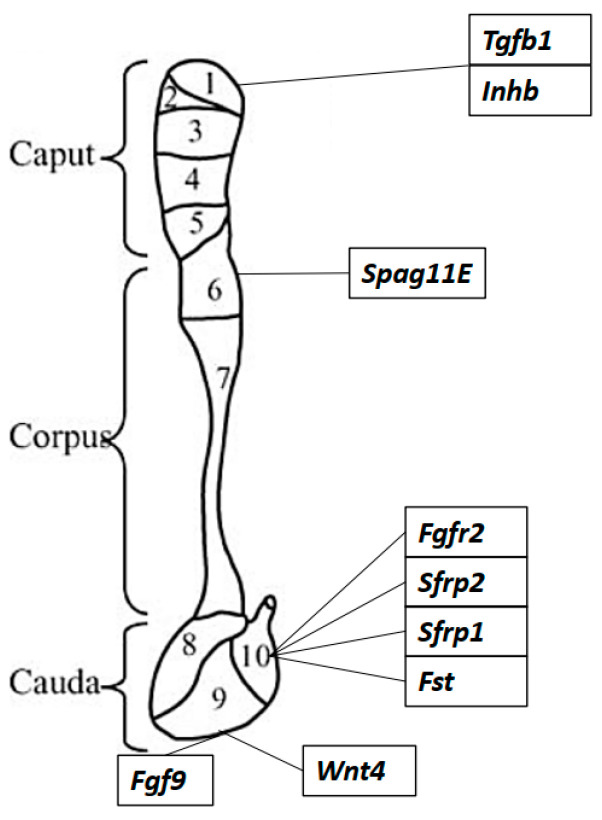
The spatial expression of coiling genes in the murine epididymis. The epididymis in mice is often divided into 10 segments based on the distinct transcriptome along the length of the epididymis. Key genes thought to be involved in coiling and epididymal convolution are listed next to the segment at which they are most highly expressed.

A study on follistatin 288 (FST288), an activin-binding protein that inhibits activin signalling, provided further evidence for activin involvement in duct coiling. In mice deficient in the FST288 isoform of follistatin, there was a 50% decrease in serum follistatin and activin A, while activin B serum levels were significantly increased [73,74]. These mice exhibited abnormal epididymal histology, reduced epididymis weight, decreased corpus duct and lumen diameter, and the ectopic coiling of the proximal vas deferens. Interestingly, follistatin expression in the vas deferens of WT mice was significantly higher than in the proximal regions of the epididymis, while activin A subunit expression showed the opposite pattern [73,74]. This highlights the role of follistatin in restraining activin activity in the vas deferens and that the dysregulation of activin A is likely to be responsible for the ectopic coiling of the vas deferens in follistatin hypomorphic mice. Ultimately, multiple signalling pathways are likely to underpin the tissue-intrinsic mechanisms of coiling in the epididymis (Figure 4).

#### 1.1.5. A Role for the Extracellular Matrix?

It is reasonable to speculate that the extensive proliferation of the tubule epithelium results in paracrine signalling to the mesenchymal cells that stimulates the mesenchyme tissue to remodel and expand, accommodating for the elongating tubule. In this concept, coiling would not be dependent on the mechanical force from the surrounding mesenchyme, and the genes and pathways mentioned earlier would more actively contribute to the coiling of the tubule. Providing further support for this theory is the fact that cells appear to proliferate at the same rate along the length of the duct, while coiling progresses caudally and occurs at specific points in the duct, suggesting that proliferation and coiling are independently regulated [2].

The role of ECM remodelling and cell–ECM interactions has been increasingly demonstrated as crucial for the morphogenesis of tubular organ structures [75,76,77]. Interactions between epithelial and endothelial cells and the ECM and the degradation of ECM components by proteases such as metalloproteinases, adamalysins and meprins, facilitate cell proliferation, differentiation, migration, adhesion and survival. The activity of these enzymes is strictly regulated by endogenous inhibitors of ECM proteinases (e.g., TIMPs, cystatin C) [75]. The ECM acts as a reservoir of growth factors, including the epithelial growth factor and fibroblast growth factor, which are released via the proteolytic activity of certain matrix metalloproteinases (MMPs) [75]. The proteolysis of ECM constituents also ensures the proper structure and composition of the matrix, and strict regulation is required to ensure proper balance between the degradation and deposition of ECM components [75]. The dysregulation of ECM deposition results in ECM stiffness as seen in fibrosis [78,79,80], while the excessive degradation of ECM components results in tissue destruction as in osteoarthritis [81,82,83]. Presumably, the remodelling of the connective tissue and ECM in the developing epididymis is a crucial part of duct morphogenesis, and the correct balance of ECM degradation and ECM stiffness is likely to play a part in facilitating the coiling of the epididymis within the mesenchymal tissue.

Notably, in mutants with a partial loss of LGR4, adult mice exhibit shorter, less coiled epididymides, multilamination of the basement membranes, and an accumulation of laminin in the mesenchyme layers of the caput region, indicating that defective ECM remodelling may contribute to the epididymis phenotype [84]. In rodent models of impaired epididymis coiling, ECM defects typically have not been considered, presenting the possibility that the observed impairments in tubulogenesis are caused by unidentified abnormal ECM remodelling, particularly in cases where cell proliferation appears to be normal. Thus, investigations of impaired epididymis elongation and coiling would benefit from consideration of the role of ECM remodelling in epididymis morphogenesis.

Proper Wolffian duct development from the intermediate mesoderm is a crucial event that prompts further urogenital ridge development, including the formation of ureteric buds [85]. Normally, Wolffian duct progenitors migrate along the anterior–posterior axis of the intermediate mesoderm, towards the cloaca, which stimulates the differentiation of the intermediate mesoderm into the nephrons of the kidneys. This highlights the importance of understanding Wolffian duct development, as its proper morphogenesis has significant consequences for other aspects of embryonic urogenital ridge development, including kidney morphogenesis. Indeed, certain factors implicated in the abnormal Wolffian duct or epididymis development are also implicated in kidney defects, although interestingly, many of the genes that drive epididymal coiling lead to kidney disease when disrupted; however, not to the aberrant coiling of the proximal or distal tubules. Autosomal dominant polycystic kidney disease (ADPKD) patients, for example, often also present with male reproductive tract irregularities and infertility [86]. In addition to renal cysts, male ADPKD patients commonly suffer from cysts in the reproductive tracts and necrozoospermia, leading to the suggestion that genes involved in ADPKD are also involved in the development and maintenance of the reproductive system. Eighty-five percent of ADPKD cases are due to mutations in the *Pkd1* gene and the remaining 15% of cases are due to mutations in the *PKD2* gene [86]. *Pkd1* KO mouse models have shown that this gene is required for normal male reproductive tract development and the production of normal sperm. *Pkd1* has been found to be expressed in the mesonephric tubules and Wolffian duct by E11.5–E12.5 in mice [86] and in 5–6-week-old human embryos [87]. *Pkd1*-deficient mice exhibited dilated efferent ducts as early as E15, which became more severe by E16.5, and the basement membrane in the efferent ducts was thickened and laminated [86], although coiling defects were not described. Cell proliferation was upregulated in the efferent ducts of KO mice compared to WT controls. Conversely, in the epididymal epithelium and mesenchyme, cell proliferation was significantly lower compared to WTs. Furthermore, at E16.5, coiling was seen in the caput epididymis of WT mice, but in *Pkd1* KOs, the tubule remained straight. Following sexual differentiation, *Pkd1* expression shifted from the epithelium to the mesenchyme, showing a shift in expression pattern and possibly function as development proceeds [86]. The epithelium-specific KO of *Pkd1* resulted in the same efferent duct and epididymis phenotype as global *Pkd1* KO mice.

This efferent duct and epididymis phenotype was also seen in global *Pkd2* KO mice and epithelium-specific *Pkd2* KOs in conjunction with the impaired development of the testes and seminal vesicles [88]. As in *Pkd1* KOs, cell proliferation was decreased and tubule coiling was absent in E16.5 *Pkd2* mutants. In mice, *Pkd2* expression in the Wolffian duct begins at E13.5 [88], while in humans, *PKD2* is expressed in the mesonephric tubules and Wolffian duct of 5–6-week-old embryos [87]. Epithelium-specific *Pkd2* KO mice exhibited impaired WNT signalling [88], indicating that epithelial *Pkd2* may directly or indirectly regulate WNT signalling or its pathway mediators. Furthermore, *Pkd1* and *Pkd2* mutant mice both exhibited impaired transforming growth factor β (TGFβ)/bone morphogenetic protein (BMP) signalling in the reproductive tract [86,88]. TGFβ/BMP signalling plays a role in the maintenance of the epididymis, as evidenced by the degeneration of the epididymal epithelium in the corpus of *Bmp4^+/−^* heterozygotes [89], in the initial segment of *Bmp7^+/−^ Bmp8a^−/−^* mutants, and the distal caput and cauda of *Bmp8a^−/−^* null mutants [90,91].

Finally, *Lgr4* encodes a receptor for R-spondins that activates the canonical WNT pathway, and is another gene implicated in both cyst formation and the abnormal development of the kidneys [1,92,93]; as well as abnormalities of the male reproductive tract. *Lgr4* KO male mice were found to be infertile, and their epididymal duct was dilated, cystic and failed to elongate and coil, presumably largely due to the observed decrease in epithelial cell proliferation [94]. In addition, the mesenchyme mass was increased and epithelial height was reduced compared to WTs. The epithelium lacked the normal pseudostratified columnar epithelial structure; instead, a bilayer of nuclei was observed in the caput and corpus, indicating the potential impairment of cell elongation and nucleus migration. Notably, the epididymis phenotypes of *Lgr4* KOs and WTs were comparable at birth, and the abnormalities in the mutants were only observed from P3 onwards, suggesting a postnatal role for LGR4 [94]. However, it should be noted that in this study, in contrast to previous reports, the kidneys of the *Lgr4* KOs were morphologically normal, and the role of LGR4 in embryonic Wolffian duct development remains to be investigated. Hypomorphic *Lgr4* mutants that have only 10% of normal *Lgr4* mRNA expression exhibit a similar phenotype; epididymides are dilated and less coiled.

**Figure 5:**
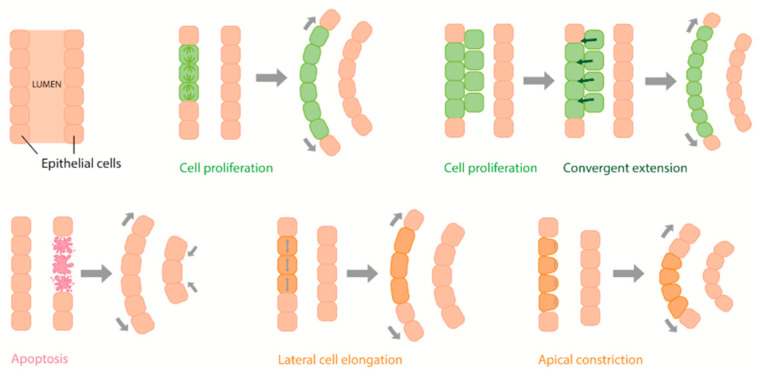
Hypothetical mechanisms of Wolffian duct and epididymal tubule coiling. Combinations of these are also possible.

One can speculate that duct coiling is the result of increased cell proliferation, convergent extension, cell death and/or cell shape changes occurring more in one side of the tubule wall than in the other (Figure 5). However, it has also been suggested that it is the space restriction itself that encourages the coiling of the elongating epididymal duct, and that the relative lack of proliferation of the mesenchyme in comparison to rapidly proliferating areas of the tubule essentially forces the duct to coil [30]. By this theory, the genes and pathways mentioned above are involved in duct elongation rather than coiling per se, with the mechanical constraint of the mesenchyme being the major driver of coiling.

### 1.2. Seminiferous Tubules

The seminiferous tubules of the testis stem from cords formed during embryonic development. From E11.5, three key events drive testis cord formation: (1) the polarisation and assembly of SOX9-positive pre-Sertoli cells to surround the developing germ cells; (2) an influx of endothelial cells from the mesonephros to form the XY vasculature and assist with the segregation of the cords; and (3) the peritubular myoid cells surround the Sertoli cells and lay down a basement membrane [3,95,96,97]. The cords originate as parallel transverse loops separated by interstitial cells [3], which then must remodel and elongate to form the seminiferous tubules. There are several excellent reviews of testis cord formation [98,99,100,101] and so we will focus this discussion on the transition from cord to tubule.

Three-dimensional modelling has recently been used to analyse testis cord development and elongation. The use of a fluorescently labelled secondary antibody that produced a uniform signal throughout the developing organ allowed the collection of high-resolution optical sections of whole-mount testes stained with this antibody to construct 3D models of foetal testes [3]. This modelling suggested that, as the testis cords begin to remodel and elongate, physical pressure pushes cords that were initial located at the edge of the organ to a more central or internal location. This study also demonstrated that testis cord remodelling occurs differently across distinct parts of the gonad; cords located on the coelomic side are organised into looped tubular structures whereas cords on the mesonephric side are coalesced into flattened structures [3]. Ultimately, testis cord formation alone is not enough, and additional factors are required to maintain and remodel these cords.

A role for activin is conserved between the testicular and epididymal tubule coiling. No testis cord coiling or convulsion occurs in the absence of activin A and interestingly the activin signal comes from outside the testis cord. When *Inhba* is deleted from foetal Leydig cells using the *Amhr2*-Cre mouse line, normal testis cord coiling is blocked between E17.5 and E19.5, resulting in only minimal remodelling of the testis cords [102]. Histological analysis of these testes revealed a significant enlargement of testis tubule diameter in the Leydig cell KO animals compared to litter mate controls and straight lengths of testis tubules were seen emerging from the rete testis, a phenotype not seen in control animals [102]. This effect was confirmed to be the result of activin A signalling to the Sertoli cells, as the phenotype was mirrored in the Sertoli cell-specific *Smad4* KO mouse line [102]. SMAD4 is a central component of canonical TGFβ signalling pathway and while other TGFβ ligands are expressed in the embryonic cords (including activin B, various TGFβs, and anti-Müllerian hormone), cord dysgenesis has not been observed in mouse models lacking these ligands.

The cell proliferation analysis of both the Leydig cell *Inhba* and Sertoli cell *Smad4* KO mice revealed that the inhibition of Sertoli cell proliferation was the likely cause of the cord-to-tubule block. Significantly reduced numbers of Ki67-positive Sertoli cells were seen in both mouse lines [102], with the reduction in proliferation more pronounced for the *Smad4* KO mice. This lack of foetal Sertoli cell proliferation resulted in testis dysgenesis in adulthood as tubule diameters remained enlarged in KO mice at 16 weeks of age. Interestingly, some tubule cross-sections containing morphologically normal seminiferous epithelia were evident in both the *Inhba* and *Smad4* conditional KO mice, suggesting that some coiling had taken place; however, there were also tubule cross-sections displaying aberrant spermatogenesis (failures of spermiation, meiotic errors, mixed-stage tubules, loss of germ cells) [102]. Taken together, these results demonstrate that activin A from Leydig cells signals to Sertoli cells to trigger proper cord remodelling to a tubule structure and that this remodelling is driven by the rapid proliferation of foetal Sertoli cells.

#### Intestine

During development, the gut tube forms directly from the endoderm, and the intestines specifically arise from the mid and hindgut. In this respect, their development differs from the epididymis and kidney in that there is lack of an intermediate structure such as the mesonephros/metanephros. The intestines undergo extensive morphological changes in order to fit almost 9 m of gut tissue within the abdominal cavity. These changes primarily occur from the sixth week of foetal development onwards [103]. Initially, the midgut protrudes through the proximal region of the umbilical cord, in a process known as physiological umbilical herniation. The protruding gut tube forms a loop, and rotates 270 degrees around the superior mesenteric artery, such that the cranial limb of the loop is positioned below the caudal limb [104]. During this process, the cranial limb forms coils which eventually become the duodenum, jejunum and ilium of the small intestine. The now coiled cranial limb descends toward the hindgut, while the caudal limb retreats from the umbilical space, takes on a vertical orientation (forming the ascending loop of the intestines), and elongates [104]. The result is a midgut composed of an obliquely orientated and coiled proximal section, followed by ascending and descending sections. This complex coiling process relies on a sophisticated series of well-timed movements in order to produce a densely coiled and thus effective digestive tract.

The importance of this sequence of rotation and coiling is demonstrated in the pathologies that result when this process is disrupted. Intestinal malrotation occurs primarily among infants when the coiling sequence of the intestines does not proceed correctly, and the results of this include life-threatening conditions including volvulus (a blockage resulting from the intestines twisting back on themselves) which can lead to tissue necrosis when blood supply is lost [105]. Defects in the mesentery, which binds parts of the gut to each other as well as to the walls of the peritoneal cavity, have been identified as contributing to the incidence of volvulus due to the incorrect rotation of the intestines.

Left–right asymmetry is important in gut formation to the extent that, along with other congenital abnormalities, its disruption may lead to intestinal malrotation as the development of the gut relies on rotation around the left–right axis [106]. Asymmetric traits in the developing mesentery are the product of asymmetric gene expression, in particular the gene *Nodal* [107]. *NODAL* is expressed on the left side of the lateral plate mesoderm and acts upstream of the homeobox gene *Pitx2*. *NODAL* is an important effector of asymmetry, regulating the gene *Foxf11* to bind to enhancers in the last intron of the *Pitx2* gene. PITX2 itself is restricted to the left side of the developing dorsal mesentery, and its expression leads to changes in both cell and tissue morphology. In the presence of PITX2, cells in the murine dorsal mesentery take on a columnar shape, as opposed to the cuboidal cells which appear in the absence of PITX2 [107]. Cells on the left also aggregate, whilst those on the right expand, and these properties in the developing dorsal mesentery lead to a leftward tilt that influences the rotation of the developing midgut [107]. NODAL expression is, however, transient, and the continued expression of PITX2 is owed to NKX2, which ensures that PITX2 continues to be expressed following the cessation of NODAL expression [108]. The regulation of dorsal mesentery by NODAL and other genes represents a critical external influence on the rotation and coiling of the developing gut.

Examining coiling processes in the intestines allows for the identification of genetic pathways and morphological processes common to embryological tissues that undergo coiling, although intriguingly, few conserved mechanisms have been determined to date. The kidneys and epididymis are derived from common embryological precursors, and as such, commonalities in gene pathways and morphological events in their development may be attributed to this common origin. The intestines, conversely, develop directly from the hindgut endoderm, and consequently, genes common to coiling in both the intestines and the epididymis/kidneys are likely to be important conserved regulators of coiling irrespective of the preceding germinal structures from which they arise.

Previous experiments to determine gene expression patterns, such as in situ hybridisation analysis of dorsal mesentery tissue, revealed that WNT5 is differentially expressed in the left side of the dorsal mesentery [109], that PITX2 drives the expression of WNT5 in the dorsal mesentery, and that genes upstream of WNT5 contain PITX2 binding sites [109,110,111]; moreover, DAAM2, for example, is a downstream target of PITX2, as well as the WNT pathway [109]. This suggests that WNT signalling as well as NODAL expression can influence PITX2 and DAAM, which in turn are necessary for the aggregation of cells on the left side of the dorsal mesentery.

The WNT pathway may also contribute to the extension of the intestines, as mutations affecting WNT5A result in a shorter gut in mice [111]. However, whether the mechanism and gene pathways influenced by WNT5A in intestinal elongation are consistent with the established role of the WNT pathway in convergence–extension type functions elsewhere in the developing embryo is not clear. Although Wnt-9b^−/−^ mouse embryos display coiling defects in the Wolffian duct, Wnt-5a^−/−^ embryos display defective intestinal phenotypes; however, these defects are characterised by the shortening of the intestines rather than defects within the coiling process itself [111]. Despite this overlap, however, there is little evidence suggesting interaction regarding NODAL and WNT5 or any of its upstream effectors.

These examples provide evidence that the coiling events which occur throughout the developing embryo possess common upstream regulators, while the majority of downstream effectors may be tissue specific.

## 2. Conclusions

There is an array of genes, proteins and pathways that contribute to the regulation and coordination of the Wolffian duct and epididymis coiling. SPAG11C is an androgen-regulated β-defensin required for Wolffian duct coiling, however, epithelial AR signalling has been shown to be non-essential for coiling. WNT signalling has recently been implicated in Wolffian duct coiling, and SFRPs are also suspected to be required for normal coiling due to their interaction with WNT receptors. *Pkd1*, *Pkd2* and *Lgr4*, genes involved in cystic kidney disease are now also known to play a role in Wolffian duct coiling. Particularly fascinating are *Lgr4* KO mice, which exhibited normal epididymides at birth with defects observable only three days later, indicating that aberrant development is restricted to the postnatal stage.

Paracrine signalling facilitated by mesenchyme-secreted proteins is likely important for the coordination of coiling. Activin A and its subunits are required for duct elongation and coiling, and activin A requires regulation by follistatin to inhibit ectopic duct coiling. Understanding epididymis development and tubule coiling will not only contribute to our understanding of male infertility, but can also provide a model for the morphogenesis of other tubular or coiled tubular organs.

Furthermore, comparison of the gene pathways and mechanisms involved in coiling in the epididymis with developmentally separate coiled tissues such as the intestines, provide the basis for the exploration of genes involved in coiling more generally throughout the embryo. In this review, we provided an overview of the factors implicated in Wolffian duct and epididymis coiling so far, and provided a brief comparison with factors governing coiling elsewhere. Although the Wnt/PCP pathway is clearly a highly conserved mechanism governing coiling, bending and shaping, few other hierarchical pathways appear to show such considerable conservation across different organs.

While there is considerable work ahead to understand how these factors interplay to organise and drive such a complex developmental event, it is clear that coiling is crucial for normal epididymis development and its function in sperm maturation, and ultimately, for fertility. Intriguingly, these genetic mechanism and androgen signalling do not substantially impact coiling in other tissues such as the intestines, suggesting that the process of coiling itself may be more to do with environmental signals, germinal layer origin and perhaps function, than with intrinsic core genetic signatures of the cell.

The task of uncovering the enigma of epididymal, intestinal, kidney tubule and coiling would benefit from a broader approach investigating additional cellular, hormonal, environmental, mechanosensitive and ECM remodelling processes to reveal impairments in the mechanisms behind defective elongation and convolution across the broad spectrum of organ formation.

## Data Availability

Not applicable.
